# Comparing the prolonged effect of interval versus continuous aerobic exercise on blood inflammatory marker of Visfatin level and body mass index of sedentary overweigh/fat female college students

**DOI:** 10.3934/publichealth.2019.4.568

**Published:** 2019-12-16

**Authors:** Alireza Moravveji, Mansour Sayyah, Elham Shamsnia, Zarichehr Vakili

**Affiliations:** 1 Department of Community and Preventive Medicine, Social Determinant of Health (SDH) Research Center, Kashan University of Medical Sciences, Kashan, Iran; 2 Departement of Exercise Sciences, Kashan University of Medical Sciences, Kashan, Iran; 3 Islamic Azad University, Kashan, Iran; 4 Department of Pathology, Kashan University of Medical Sciences, Kashan, Iran

**Keywords:** aerobic exercise, visfatin, overweight, student

## Abstract

**History and objectives:**

Over weightiness and obesity are usually defined as inflammatory conditions. High ratio of body mass index and Visfatin level recently discovered as markers involved in inflammatory process of obesity. Aerobic exercise is one of the safe interventions to decrease such condition. The purpose of this research was to compare the effect of interval versus continuous aerobic exercise on Visfatin and BMI of sedentary overweight female college students.

**Materials and Methods:**

Thirty-six healthy sedentary overweight female college students with BMI over 25 or more were randomly assigned into three groups including continuous, interval aerobic exercise and control conditions for eight weeks, three sessions per week. Serum visfatin level was assessed before and after the exercise protocol. The exercise protocol included running a distance of 1200 meters continuously or with rest intervals at 60 to 75 percent of reserved heart rate in the first week that gradually increased by 400 meters on every subsequent week.

**Results:**

Our study indicated that both aerobic exercise conditions significantly decrease the serum level of visfatin (P = 0.000, P = 0.025, respectively). Both exercise groups also showed a decrease in BMI compared to the control group (P = 0.006, P = 0.004).

**Conclusion:**

Aerobic exercise has a beneficiary effect on both serum visfatin level and BMI variables involved in inflammation process of obesity regardless of being performed with rest interval or continuously.

## 1. Introduction

Obesity is one of the known health risk factors with high prevalence that rises steadily worldwide by many factors including high calorie diets, use of motor devices and looking at some sorts of screens such as mobile set instead of taking part in regular physical activities. All these factors lead to over weightiness or obesity, a major risk factor for cardiovascular disease [1]–[5], diabetes, hyperlipidemia, and atherosclerosis [6]–[8]. The results of several studies show that there is an association between adiposity and increased risk of hypertension, insulin resistance, diabetes mellitus, dyslipidemia all causing increase in the risk of myocardial infarction [9]–[12].

Regular aerobic exercise program can potentially modify metabolic hormones and is considered as an important treatment of chronic inflammation and obesity-related conditions [13]. However, there are some controversies on such benefits which also may vary with the type and amount of exercise. A systematic review of the effect of chronic exercises on inflammatory factors including leptin and adiponectin concentrations released from fat tissue in adults revealed contradictory findings [14]. Also, a recent meta-analysis in patients with type-2 diabetes showed that aerobic exercise program was associated with a significant change in leptinbut did not alter adiponectin levels [15]. Furthermore, a review on pediatric obesity indicated that exercise has an impact on the adipose tissue and the release of adiponectin, resistin, and visfatin [16].

Aerobic exercise may be performed with rest interval or continuously. An interval aerobic exercise is performed with rest intervals interspaced between several bouts of aerobic activities whereas continuous aerobic exercise is performed for certain period of time without any rest up to the point of completion. Thus, despite the fact that regular aerobic exercise program remains as a preventive and treatment mean to deal with obesity incidence and cardiovascular diseases [17], there seems to be a need to conduct more researches about the effectiveness of different types of aerobic exercise programs. In addition, considering more discoveries about the fat tissue and its products involved in inflammation, we should determine more beneficial types of aerobic exercise programs in changing the level of inflammatory factors such as visfatin released by fat tissue [18] in susceptible populations to obesity such as college student girls [19]. Visfatin, as a novel adiponectin identified in 2005, plays a significant role in the regulation of insulin production [20]. There are researches that show visfatin level increases with obesity [21]. In regard to the effect of aerobic exercise on visfatin level, controversial results have been reported. While a research results showed that 12 weeks of aerobic exercise did not significantly altered visfatin level of the participants [22], another researcher reported that aerobic exercise did change the level of visfatin in healthy men [18]. Considering these contradictory findings, the present study was designed, for the first time, to compare the effect of two types of aerobic exercise of interval versus continuous on serum visfatin level and BMI of overweight or obese college girl students.

## 2. Materials and methods

The participants: The participants were 36 college girls divided randomly divided into three groups and then randomly assigned into the groups of 12 girls per group though the use of random number table. They were volunteered to take part in the research. They learned about the protocol through the advertising placed in college campuses and dormitories. The inclusion criteria consisted of having Body Mass Index (BMI) over 25, not being involved in any regular physical activities for the past year, and being in a good healthy condition. The individuals with BMI less than 25, having a recent history of participation in regular physical activities such as being a sport team member or registered in sport clubs, having health problems such as high blood pressure and diabetes were excluded. BMI was calculated by dividing the weight in kilogram to the square of height in meter.

Experiment: One day prior to the start of the exercise protocols, all participants attended a pathologic lab where a 5 cc of venous blood was drawn. Plasma visfatin concentrations were measured by enzyme linked Immune Sorbent assay (Elisa) kit (eBioscience, USA) according to manufacturer's instructions.

Exercise protocol: The participants took part in session to receive instruction about how to perform the exercise program before the start of the protocol. The exercise groups performed the pretest exercise program, but the control group was asked to do their regular daily works and stay away from participating in any regular exercise programs. The exercise protocol included 5 minutes of warm up followed by the running exercise. The exercise program was performed three times perweek. Every session was scheduled in the afternoon. The control group did not participate in the exercise protocol.

The exercise started at 60 to 75 percent of reserved heart rate set by polar watch (China made, t-31) for a distance of 1200 meters in the first week without rest. The work intensity was based on 60 to 75 percent of maximum heart rate (max hr = 220-age). Thus, every participant exercised at approximately similar intensity. The participants were instructed to maintain their work intensity at the defined work level. Every week, 400 meters more distance was added to the distance of 1200 meter running program [34]. The interval exercise program included running 1200 meter; three runs of 400 meter ( 3 * 400 = 1200) interspaced by a walking period of two minutes whereas the continuous exercise group ran the entire 1200-meter distance without any rest intervals. Every week, a distance of 400 meter was added to the running distance to avoid adaptation to the exercise program. Overall, five participants dropped from the exercise protocol. Three of the participants from the control group terminated their participation and were not willing to continue their cooperation. Two participants were unable to continue their work due to injury; one to the ankle twist and the other due to knee problem. Finally, 10 participants in the continuous, 12 in the interval exercise and 9 in control groups respectively completed the research project.

Statistical analysis: Kolmogrove-Smirinov test was used to evaluate the distribution of the dependent variables. The result indicated that all the independent variables were normally distributed and parametrical statistical tests were used to analyze the data. One-way ANOVA was used to test the hypothesis. All analyses were performed by using SPSS: PC 16.0. The level of significance was set to alpha equal to 0.05.

## 3. Results

**Figure 1.**
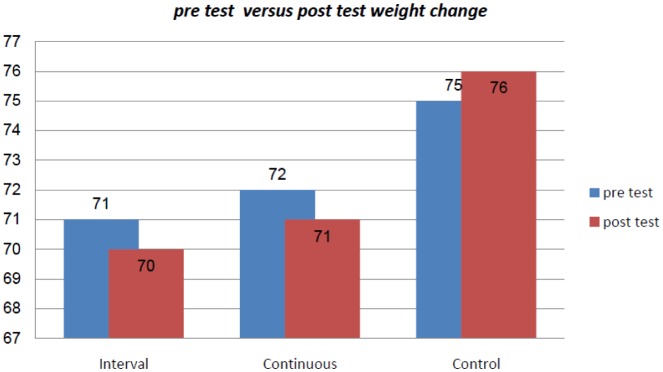
Changes in body weight(kg)from the pre test to post test state.

**Figure 2.**
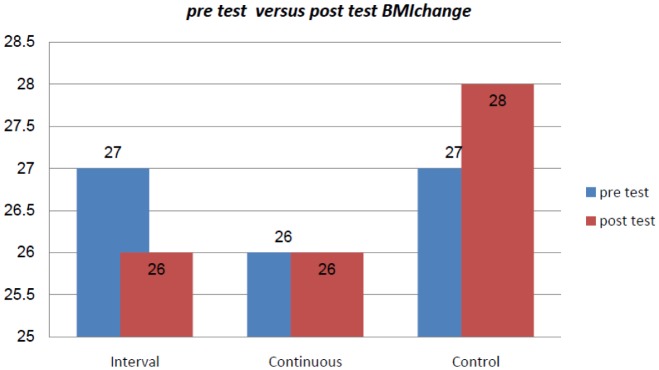
Changes in BMI(kg/mm) from the pre test to post test state.

There were not any significant differences among the interval, continuous exercise and control groups in regard to age, weight, BMI and serum visfatin level (P = 0.97, 0.26, 0.19, 0.57, respectively at the start and end of the exercise protocol (table 1). There was not any significant difference between the independent variables between the three groups before the exercise protocol. Analysis of covariance (ANCOVA) showed that BMI of the participants in pretest condition was not significantly associated with visfatin changes after the termination of the exercise protocol (P = 0.603) (data not shown). The result showed that there was a significant difference in weight (P = 0.01), BMI (P = 0.005) and visfatin (P = 0.00) level among the three groups of interval, continuous exercise and control group after the exercise protocol. The changes in body weight and BMI from pre test to post test state are presented in Figure 1 and 2.

**Table 1.** Basic statistics of age, weight, BMI and visfatin level of blood serum in pretest and post test stage for the interval, continuousand control groups.

Variables	Group	Mean±SD (pretest)	Sig.	Mean±SD (posttest)	p-value
Age (years)	Interval	20.7 ± 2.71	0.97	-	-
	Continuous	20.33 ± 1.49		-	
	Control	20.66 ± 2.24		-	
Weight (kg)	Interval	71.10 ± 5.40	0.26	70.10 ± 4.67	0.01
	Continuous	72.75 ± 6.18		71.25 ± 5.57	
	Control	75.33 ± 5.72		76.88 ± 5.37	
BMI (kg/mm)^2^	Interval	27.57 ± 1.02	0.19	26.82 ± 0.98	0.005
	Continuous	26.77 ± 1.53		26.08 ± 1.21	
	Control	27.57 ± 1.62		28.04 ± 1.46	
Visfatin (ng/ml)	Interval	49.37 ± 16.68	0.57	30.92 ±10.74	0.001
	Continuous	56.32 ± 13.9		43.03 ± 8.01	
	Control	51.07 ± 17.3		52.14 ± 6.79	

For the significant differences that were found, post hoc multiple comparisons test was used to locate the differences among the means by using LSD method. These are presented in table 2. LSD test result indicated that there was a significant decrease for weight for both exercise groups compared to the control group (P = 0.01, 0.02), but there was no significant difference between the continuous and interval exercise groups (P = 0.61). LSD post hoc test was used to determine the significant differences between the BMI for the three groups; the result showed that the significant differences was between the interval and continuous group compared to the control group (P = 0.006, P = 0.004). No significant difference was found between the interval and continuous exercise group (P = 0.96). Post hoc test using LSD method also confirmed such significant difference between the visfatin serum level of interval and continuous group compared to the control group (P = 0.003, P = 0.025). Also, there was a significant difference between the rest interval and the continuous group (P = 0.003).

## 4. Discussion

The purpose of this research was to compare the effect of two types of aerobic exercise on serum visfatin (as an inflammatory marker of adipose tissue) and weight as well as BMI (as markers of inflammatory condition of obesity) of fat sedentary college girls. These variables were measured before and after the participation of the students in a progressively increasing exercise program either continuous or with rest intervals. Our results showed that both exercise types significantly decreased the level of inflammatory marker of visfatin, weight and BMI compared to the control group.

From the time of discovery of the first adiponectin, leptin, fat tissue has been identified as an endocrine organ that plays a significant role in energy storage [23],[24]. Newly discovered adiponectin, visfatin, has been identified as a new adipokine preferentially produced in the visceral adipose tissue of obese mice and humans [25].

**Table 2.** LSD post hoc test for weight, BMI, and visfatin comparing interval, continuous exercise and control group.

Variable	Group	Group	Mean Difference (I-J)	Std. Error	Sig.
Weight(kg)	interval	continuous	−1.15	2.28	0.618
		control	−6.78*	2.44	0.010
	continuous	interval	1.15	2.28	0.618
		control	−5.63*	2.35	0.023
	control	interval	6.78*	2.44	0.010
		continuous	5.63*	2.35	0.023
BMI(kg/mm)	interval	continuous	0.73	0.814	0.986
		control	−1.21*	0.52	0.174
	continuous	interval	−0.73	0.56	0.040
		control	−1.94*	0.52	0.174
	control	interval	1.21*	0.54	0.001
		continuous	1.94*	0.56	0.040
Visfatin(ng/ml)	interval	continuous	−12.11*	0.54	0.001
		control	−21.22*	3.99	0.000
	continuous	interval	12.11*	3.72	0.003
		control	−9.11*	3.83	0.025
	control	interval	21.22*	3.99	0.000
		continuous	9.11*	3.83	0.025

Note: * The mean difference is significant at the 0.05 level.

Aerobic exercise is a common interventional method that leads weight loss and may be performed intervallic or continuously. Considering BMI as a serious health risk factor which increases nearly 4-fold higher risk of heart failure and 2-fold higher risk of stroke attack [5], we conclude that some beneficiary effects of aerobic exercise may acts through the decrease of visfatin which in turn decreases the BMI.

The results of the present research were similar to those of several studies that showed plasma visfatin decreases in patients with type 2 and 1diabetic patients after aerobic exercise [26]–[29]. Also, it was showed that six weeks of swimming exercise significantly decreased the serum level of visfatin in rats [30]. These findings are also similar to those of some others which examined the effects of aerobic exercise on visfatin level in over weight populations [27]. For example, 12 weeks of aerobic exercise in diabetic obese women resulted in a decrease of visfatin level; 12 weeks of aerobic exercise performed at 60 to 75 percent of maximum heart rate significantly decreased the serum level of visfatin of sedentary obese women aged between 30 to 55 [30].

Considering the fact that obesity prevalence is on the rise worldwide [31], and it has undesirable consequences on health matter even in sport populations [32], every measure including the use of different types of physical activity, particularly, aerobic exercise regardless of being performed intervallic or continuously are effective intervention to control or prevent it. These types of exercises also have beneficiary effect on variables involved in inflammation process. Visfatin can exert various deleterious effects on vascular cells, including inflammation and proliferation [33].

Finally, the researcher admits the limitation of this study. More research including larger sample size, male gender, longer periods of exercise and other modes of exercise are needed to make firm recommendations.

## 5. Conclusion

Based on our findings, we may conclude that both types of interval or continuous aerobic exercise are effective in decreasing over weightiness and visfatin level, inflammatory markers that is a health risk factor. Aerobic exercise with rest or without rest interval has beneficiary effect on health risk factors.
